# The first complete chloroplast genome of a traditional Chinese medicinal herb *Odontosoria chinensis* (Lindsaeaceae)

**DOI:** 10.1080/23802359.2018.1443045

**Published:** 2018-02-26

**Authors:** Ruixiang Xu, Shanshan Liu, Zhen Wang, Ting Wang, Yingjuan Su

**Affiliations:** a School of Life Sciences, Sun Yat-sen University, Guangzhou, China;; b College of Life Sciences, South China Agricultural University, Guangzhou, China;; c College of Life Sciences, Nanjing Agricultural University, Nanjing, China;; d Research Institute of Sun Yat-sen University in Shenzhen, Shenzhen, China

**Keywords:** *Odontosoria chinensis*, chloroplast genome, traditional Chinese medicinal herb, phylogenetic analysis

## Abstract

It is the first report on complete chloroplast genome of *Odontosoria chinensis*, a traditional Chinese medicinal herb. The genome size is 156,293 bp, containing a pair of inverted repeats (IRs) (25,198 bp) separated by a small single-copy (SSC) region (20,649 bp) and a large single-copy (LSC) region (85,248 bp), respectively. The plastome has 128 genes, including 87 protein-coding genes, 32 tRNA genes, eight rRNA genes, and one pseudogene. The overall GC content is 40.43%. Maximum likelihood tree reveals that *O. chinensis* is basal lineages of polypods. The study will provide powerful molecular information for further phylogenetic analysis.

*Odontosoria chinensis* (L.) J. Sm., commonly known as *Sphenomeris chinensis* (L.) Maxon, belongs to Lindsaeaceae and is widespread throughout southern China at higher altitudes in evergreen forests (Dong et al. 2013). It is a perennially terrestrial herb with short-creeping rhizomes, pale brown stipes and rachises, and narrowly obovate ultimate segments (Dong et al. 2013). Except as a beautiful ornamental plant, the species is also a traditional folk medicine in China with reputation as an “all-purpose antidote” for dysentery, food and pesticide poisoning, burn injury, and incised wound (Jiangsu New Medical College 1977; The National Assembly Group of Chinese Herbal Medicine 1983). *Odontosoria chinensis* represents a key member to explore diversification of lindsaeoid ferns (Lehtonen et al. 2012) and phylogenetics and classification of Lindsaeaceae (Lehtonen et al. 2010; Christenhusz et al. 2011), acquirement of its whole chloroplast (cp) genome sequence will promote our understanding for these issues.

We collected fresh leaves of *O. chinensis* from South China Botanical Garden, Chinese Academy of Sciences (23°19’28.21’’N, 113°37’47.46’’E). The specimen is stored in Herbarium of Sun Yat-sen University (SYS; voucher: *SS Liu 20161010*). Genomic DNA was extracted using Tiangen Plant Genomic DNA Kit (Tiangen Biotech Co., Beijing, China) and then subjected to construct a pair-end library and sequencing in Illumina Hiseq 2500 platform (Illumina Inc., San Diego, CA). After the low-quality reads were filtered out and adaptor sequences were removed by Trimmomatic v0.32 (Bolger et al. 2014), approximately 1.77 G of high-quality clean data were obtained and *de novo* assembled by Velvet v1.2.07 (Zerbino and Birney 2008). The annotation was performed using the Dual Organellar GenoMe Annotator (DOGMA; Wyman et al. 2004) and tRNAscan-SE programs (Lowe and Eddy 1997) and finally validated with BLAST searches. The circular chloroplast genome map was generated using OrganellarGenomeDraw (OGDRAW) (Lohse et al. 2013). Phylogenetic analysis based on maximum likelihood was performed through RAxML v8.0 (Stamatakis 2014) using complete cpDNA of 16 ferns including *Marsilea crenata* as outgroup.

The whole chloroplast genome of *O. chinensis* is 156,293 bp in length with 40.43% GC content, and exhibits a typical quadripartite structure including two inverted repeats (IRa and IRb) of 25,198 bp each separated by a small single-copy (SSC) of 20,649 bp and a large single-copy (LSC) of 85,248 bp (GenBank accession number: MG913608). It possesses 128 genes including 87 protein-coding genes, 32 tRNA genes, eight rRNA genes, and one pseudogene (*ndh*B). Among these genes, each of IR region contains four protein-coding genes (*psbA*, *rps7*, *rps12*, and *ycf2*), five tRNA genes (*trnA-UGC*, *trnH-GUG*, *trnI-GAU*, *trnN-GUU*, and *trnR-ACG*), and four rRNA genes (*rrn4.5*, *rrn5*, *rrn16*, and *rrn23*). In addition, 12 genes (*atpF*, *ndhB*, *petB*, *petD*, *rpoC1*, *rpl16*, *rpl2*, *trnA-UGC*, *trnG-UCC*, *trnI-GAU*, *trnL-CAA*, and *trnV-UAC*) contain one intron, while three genes (*clpP*, *rps12*, and *ycf3*) have two introns. GC content of the IR, SSC, and LSC is 44.17%, 36.07%, and 39.27%, respectively. ML tree reveals that *O. chinensis* is basal lineages of polypods (Figure 1). The first complete chloroplast genome of *O. chinensis* will provide very powerful molecular information for further phylogenetic studies.

**Figure 1.**
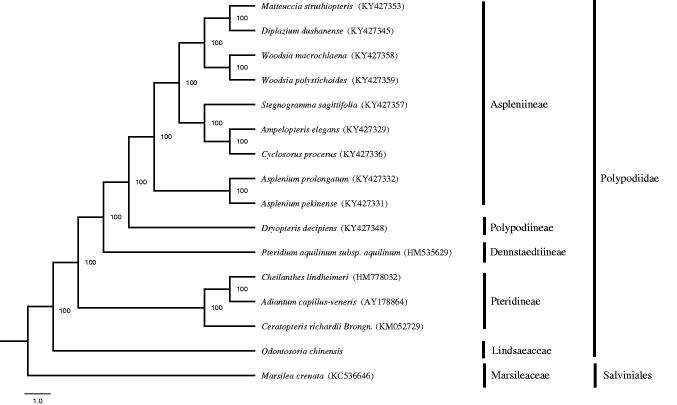
Maximum likelihood tree based on chloroplast genome sequences of 16 ferns including *Marsilea crenata* as outgroup. Numbers near branches show the bootstraps with 1000 replicates.
